# Emotional impact on the results of BRCA1 and BRCA2 genetic test: an observational retrospective study

**DOI:** 10.1186/s13053-017-0077-6

**Published:** 2017-10-02

**Authors:** Sara Mella, Barbara Muzzatti, Riccardo Dolcetti, Maria Antonietta Annunziata

**Affiliations:** 1 Unit of Oncological Psychology, Centro di Riferimento Oncologico - National Cancer Institute, Aviano, Italy; 2 Cancer BioImmunotherapy Unit, Centro di Riferimento Oncologico - National Cancer Institute, Aviano, Italy

**Keywords:** BRCA1/2, Emotions, Genetic counseling, Mood states, Psychological distress

## Abstract

**Background:**

BRCA1 and BRCA2 mutations are associated with a higher risk of breast and ovarian tumors. This study evaluated the emotional states of women 1 month after having received the results of the genetic test and assessed eventual associations with the type of outcome, personal/familiar disease history and major socio-demographic variables.

**Methods:**

The study, an observational retrospective one, involved 91 women, evaluated 1 month after receiving their results. Patients were administered the Hospital Anxiety and Depression Scale, the Profile of Mood States and emotional Thermometers.

**Results:**

Anxiety was significantly higher than depression (*p* < 0.001), and 21.3% and 21.3% of the sample were, respectively, possible and probable cases for anxiety, whereas 13.5% and 10.1% were possible and probable cases for depression. Within the six mood states, Confusion-Bewilderment (M = 48.5) was the lowest, whereas Fatigue-Inertia (M = 52.3) was the highest. Differences were recorded within the ten assessed emotions too. Being a proband/nonproband and being or not a cancer patient were associated with many tested variables.

**Conclusion:**

The psycho-emotional screening of women undertaking genetic counseling is relevant and should cover a large range of dimensions.

## Background

In Italy, breast cancer is the most common cancer, accounting for 29% of all tumors. Ovarian cancer is the tenth most frequently diagnosed cancer in women, accounting for 3% of all diagnosed cases [1].

In a minority of cases, within certain families, these diseases are more frequent than in the general population. When the recurrence of the disease or its modalities [2] leads to the possibility of a genetic predisposition being present in the family (i.e. high-risk families), the test for the identification of BRCA1 and BRCA2 (BReast CAncer gene 1 and 2) mutations is proposed. It is a genetic susceptibility test that identifies germinal and dominant autosomal inheritance increasing the risk of breast/ovarian cancer.

However, it must be borne in mind that only in 20–25% of index cases (i.e., probands) that underwent a genetic test were clear pathogenic significant mutations found (i.e., positive result, being a carrier) [3–6]. Meanwhile for 75–80% the indefinite result, negative or inconclusive (i.e., no individuation of BRCA1/2 mutations not excluding the possibility of different, still unknown mutations) or indicative of possibilities of variability still not classified, precludes the possibility of extending the test to relatives (i.e., non probands) and having available clear and consensual guidelines for risk management [7]. As a consequence, a negative result (i.e., being a noncarrier) can be interpreted differently depending on whether it refers to a proband or a proband’s relative. Only in the second case are data informative and allow to rule out the presence of the mutation previously found in the family.

Given the multifactorial nature of the disease, it is not easy to precisely measure the strength of the risk of a neoplasm occurring in the presence of a mutation. However, generally speaking, it is possible to state that individuals bearing BRCA1/2 mutations have a 40–60% risk of developing breast cancer and a 20–40% risk of developing an ovarian tumor in their life [8].

The genetic test has significant implications for women’s personal, family, and social life [9]. For this reason, it is useful to include it into a broader view of the genetic counseling field as a complex communicative action [10], in which both bio-medical and psycho-social aspects are addressed. In genetic counseling, in fact, individuals receive and face information on the possible genetic nature of their illness; face risk and uncertainty; evaluate the possibility of being included in adequate clinical-instrumental surveillance protocols or strategies for the reduction of risk that may have repercussions on their self-image; and have to consider the moral “obligation” to share this information with their relatives.

The possibility that such information may have repercussions on a psycho-emotional level has prompted studies on the evaluation of psychological distress (commonly anxiety, depression and cancer-related worries) prior to and after the interview when results are communicated [11]. These studies, which have included the percentage of individuals affected and not affected by oncological disease and with positive, negative or indefinite results [11], demonstrated that levels of distress have been associated with genetic testing decision-making, risk reduction decisions and screening adherence [12].

The literature on distress levels in individuals belonging to high-risk families is conflicting; some studies (e.g. [13, 14]) have evidenced high distress levels in respect of the general population – particularly with regard to anxiety and cancer-related distress – while others (e.g. [15, 16]) have not reported statistically significant differences between the two populations.

More specifically, for the results deriving from the genetic test, the majority of researches have described the short-term psychosocial impact of BRCA1/2 genetic testing, mostly in the year after receiving results [17]. Usually, the data at our disposal seem to exclude the presence of severe issues in individuals who are told of the presence of a predisposing mutation (carriers) [11]; however, some studies have reported anxiety and depression [18, 19], anger and distress [20, 21], cancer-related worry [22], vulnerability and stigma, and alterations in self-perception and quality of life [23, 24].

Moreover, as highlighted by Pearman [25], many studies (e.g. [26]) have reported that levels of distress do not seem to differ among affected (i.e. carriers) and unaffected (i.e. noncarriers) women by a known genetic mutation but rather seem to be associated with belonging to high-risk families.

The lack of clear answers and the resulting uncertainty can be potential sources of emotional suffering and decisional conflicts. The discrepancy among literature data – because of the complexity of all the variables considered – does not allow univocal identification of the categories of people potentially vulnerable to psychological suffering who deserve particular attention, apart from individual variability.

## Methods

### Aims

The aim of the present study was to assess the emotional state of women who underwent a genetic test for the identification of BRCA1 and 2 mutations, 1 month after the communication of their result and to verify possible associations between emotional state, test result (being a carrier/noncarrier), family history (including proband/non proband status) and major socio-demographic variables. To this end, two validated psychological instruments (Hospital Anxiety and Depression Scale; Profile of Mood States) were used, as well as an ad hoc instrument (emotional Thermometers) that allowed to widen the spectrum of emotional states, generally investigated in literature, and the eventual presence of positive emotions.

### Participants

The study enrolled consecutive women, probands (i.e. patients with breast or ovarian cancer who initiate a counseling process for their family) and nonprobands (i.e. relatives of a proband), who underwent a genetic test for the identification of BRCA1 and BRCA2 mutations, in a cancer institute in the northeast of Italy, who had received the results of their test. Other inclusion criteria, apart from being females, included: being >18 years old, a good knowledge of the Italian language, and absence of physical or sensory disabilities that may hinder the filling in of questionnaires.

All participants read and signed an informed consent form.

### Tools and procedure

The women who agreed to participate in the study received materials by mail within a month of the medical geneticist’s disclosure of their genetic test results. Before the genetic test was performed, patients had been informed on inclusion criteria, and had been illustrated the genes to be investigated and all possible outcomes. They were also briefed on the percentages of breast and ovarian cancers caused by mutations of the genes being screened and were explained the relationship between mutations and the risk of tumour onset throughout life. Patients were also illustrated hereditary mutation transmission mechanisms and were explained the test benefits to both probands and their family members (nonprobands). At the delivery of the test result, the geneticist explained its meaning and possible limitations of a negative result; especially in the case of a positive outcome, the geneticist insisted on the importance of sharing information with family members, and offered indication for intervention and / or prevention guidance.

The study materials included the Hospital Anxiety and Depression Scale (HADS; [27]), the Profile of Mood States (POMS; [28]), several ad hoc built emotional Thermometers, a form for the collection of socio-demographic and clinical data, and a prepaid envelope for returning material.

HADS is a self-assessment scale largely used in medical settings (especially in oncology) to register inpatients’ psychological distress [29, 30]. The tool is made up of 14 items, divided into two subscales (Anxiety and Depression) [31] to which the subject responds by using a four-point scale (0–3). Zigmond and Snaith [27] recommend the use of a threshold >10 for each subscale (Anxiety and Depression) in order to identify all probable cases and a threshold of >7 for all possible cases.

POMS is a widely used tool for the assessment of mood states. It consists of 58 items offering a mood state profile using five negative mood scales (Tension-Anxiety, Depression-Dejection, Anger-Hostility, Fatigue-Inertia, Confusion-Bewilderment) and one positive scale (Vigor-Activity). Participants rate each item on a 0–4-point answer scale. For each mood state, raw scores were converted into t-scores (with mean = 50 and standard deviation = 10) [28]. Higher values indicate higher mood state intensity.

The ad hoc built emotional Thermometers asked participants to grade on a 0–10-point scale the intensity of ten different emotions: anger, anxiety, concern, confidence, confusion, discomfort, fear, guilt, sadness and serenity.

The socio-demographic and clinical data form enabled the collection of information about age, education, occupational status, marital status, having children, proband or nonproband status, being or not a carrier, cancer diagnosis and being or not under cancer treatment.

This study received the approval of the Independent Ethics Committee of our Institute.

### Statistical analyses

Descriptive statistical analyses were conducted for each considered dependent variable. To test the association between the considered dependent variables (i.e. anxiety and depression as detected by HADS, the six mood states registered by POMS, and the 10 considered emotions) and the samples’ characteristics —being a proband/nonproband; being a carrier/noncarrier; being or not a cancer patient; being or not under cancer treatment, age (<49 vs. 49+ years), education (compulsory vs. post-compulsory), marital status, parity —Mann-Whitney tests were performed.

A Wilcoxon signed ranks test and two Friedman tests were performed to verify differences, respectively, in psychological distress and both mood states and emotions within subjects.

In all analyses, *p* < 0.05 (two-tailed) was considered to be statistically significant. Bonferroni correction was used to avoid errors due to multiple comparisons. The Statistical Package for the Social Sciences (SPSS) was used to perform the analyses.

## Results

### Sample characteristics

The sample included 91 consecutive participants with a median age of 48 years (range: 23–75). Seventy-four percent of the sample had a life partner (either married or cohabiting); 80.2% reported having at least one child. With regard to education, 48.3% reported having completed their compulsory education (in Italy, it is 8 years of education) whereas 51.7% had a post-compulsory education. A total of 65.9% of the sample were gainfully employed whereas 34.1% were housewives, students, unemployed or retired. In terms of status, 82.4% of the participants were probands whereas 17.6% were nonprobands. A proportion of 13.2% of the sample received a genetic diagnosis of carrier, and 86.8% that of noncarrier. A total of 87.9% of the sample were cancer patients, and 38.8% were under cancer treatment.

### Psychological distress

Table 1 displays mean scores of anxiety and depression for the entire sample and according to the statistically associated sample characteristics.

**Table 1:** Psychological distress in the entire sample and according to the associated sample’s characteristics (means and [standard deviations])

	Probands (*N* = 75)	Nonprobands (*N* = 14)
Anxiety	6.73* (3.84)	11.00 (5.07)
Depression	4.59* (3.58)	8.14 (4.29)
	Cancer Patients (*N* = 80)	No Cancer Patients (*N* = 9)
Anxiety	6.94* (4.11)	11.56 (4.10)
Depression	4.80* (3.81)	8.22 (3.42)
	Total (*N* = 89)	
Anxiety	7.40 (4.31)	
Depression	5.15 (3.89)	

*p-value was statistically significant according to Bonferroni’s correction

Anxiety was significantly higher than depression (*p* < 0.001), and 21.3% and 21.3% of the sample were, respectively, possible and probable cases for anxiety, whereas 13.5% and 10.1% were possible and probable cases for depression.

Nonproband participants displayed both higher anxiety (*p* = 0.003) and depression (*p* = 0.002) than probands; nondiseased healthy participants displayed both higher anxiety (*p* = 0.003) and depression (*p* = 0.005) than cancer patients; neither anxiety nor depression was found to be associated with being a carrier/noncarrier, being or not under cancer treatment, age, education, marital status or having children.

### Mood states

Proportions of 7.8% and 22.2% of the sample displayed a score that exceeded, respectively, 2 and 1 standard deviation from the normative mean score of 50 in Tension-Anxiety; 4.6% and 14.9% in Depression-Dejection; 8.9% and 17.8% in Anger-Hostility; 10% and 22.2% in Fatigue-Inertia; and 2.2% and 15.7% in Confusion-Bewilderment; whereas 3.3% and 21.1% displayed a score, respectively, of 2 and 1 standard deviation lower than the normative mean score in Vigor-Activity.

Table 2 displays mean scores of the six considered mood states for the complete sample and according to the statistically associated characteristics. In the complete sample, at least one of the 15 possible paired comparisons between the six mood states was statistically significant (*p* < 0.001); the subsequent qualitative examination of the profile showed Confusion-Bewilderment (M = 48.5) as being lower than the others, whereas Fatigue-Inertia (M = 52.3) was higher.

**Table 2:** Mood states in the entire sample and according to the associated sample’s characteristics (means and [standard deviations])

	Probands (*N* = 75)	Nonprobands (*N* = 15)
Tension-Anxiety	49.68 (10.65)	60.00 (15.29)
Depression-Dejection	48.62 (9.20)	56.07 (13.24)
Anger-Hostility	49.20* (9.42)	62.80 (17.92)
Fatigue-Inertia	50.72 (10.50)	60.27 (15.09)
Confusion-Bewilderment	47.19 (10.12)	54.67 (12.19)
Vigor-Activity	52.39 (11.86)	46.33 (11.67)
	Cancer Patients (*N* = 80)	No Cancer Patients (*N* = 10)
Tension-Anxiety	50.19 (11.29)	61.10 (14.37)
Depression-Dejection	48.78* (9.41)	57.80 (13.20)
Anger-Hostility	50.05* (11.32)	62.80 (14.16)
Fatigue-Inertia	51.25 (11.25)	60.80 (13.65)
Confusion-Bewilderment	47.56 (10.45)	55.50 (11.41)
Vigor-Activity	52.00 (12.04)	46.40 (10.79)
	Total (*N* = 90)	
Tension-Anxiety	51.40 (12.08)	
Depression-Dejection	49.82 (10.24)	
Anger-Hostility	51.47 (12.26)	
Fatigue-Inertia	52.31 (11.85)	
Confusion-Bewilderment	48.45 (10.80)	
Vigor-Activity	51.38 (11.98)	

*p-value was statistically significant according to Bonferroni’s correction

Nonproband participants reported a higher level of anger-hostility than proband ones (*p* = 0.004). Non-diseased participants displayed both higher depression-dejection (*p* = 0.008) and anger-hostility (*p* = 0.003) than cancer patients. No association emerged between being a carrier/noncarrier, being or not under cancer treatment, age, education, marital status or parity and any of the six considered mood states.

### Emotions

Table 3 displays mean scores of the ten considered emotions in the entire sample and according to the statistically associated sample characteristics. In the complete sample, at least one of the 45 possible paired comparisons between the ten considered emotions was statistically significant (*p* < 0.001); the subsequent qualitative examination of the data showed guilt (M = 1.18) and confusion (M = 2.47) to be lower than the other emotions, whereas confidence (M = 6.28) and serenity (M = 5.40) were higher.

**Table 3:** Emotions in the entire sample and according to the associated sample’s characteristics (means and [standard deviations])

	Probands (*N* = 75)	Nonprobands (*N* = 15)
Anger	2.25* (2.81)	5.13 (3.40)
Anxiety	3.17* (3.01)	6.13 (3.36)
Concern	4.24* (3.04)	7.13 (2.77)
Confidence	6.60* (2.24)	4.67 (1.95)
Confusion	2.09 (2.72)	4.33 (3.50)
Discomfort	2.24* (2.78)	4.67 (2.87)
Fear	3.13 (2.94)	5.07 (3.17)
Guilt	1.16 (2.27)	1.27 (1.79)
Sadness	2.88* (3.08)	5.93 (3.52)
Serenity	5.81* (2.49)	3.33 (1.95)
	Cancer patients (*N* = 80)	No Cancer Patients (*N* = 10)
Anger	2.39* (2.92)	5.50 (3.14)
Anxiety	3.31 (3.10)	6.50 (3.14)
Concern	4.36* (3.08)	7.60 (2.37)
Confidence	6.41 (2.36)	5.20 (1.40)
Confusion	2.21 (2.85)	4.50 (3.27)
Discomfort	2.39* (2.90)	4.70 (2.31)
Fear	3.24 (2.97)	5.20 (3.29)
Guilt	1.20 (2.28)	1.00 (1.25)
Sadness	2.99* (3.22)	6.60 (2.50)
Serenity	5.73* (2.45)	2.80 (2.1)
	Total (*N* = 90)	
Anger	2.73 (3.09)	
Anxiety	3.67 (3.24)	
Concern	4.72 (3.17)	
Confidence	6.28 (2.30)	
Confusion	2.47 (2.97)	
Discomfort	2.64 (2.92)	
Fear	3.46 (3.05)	
Guilt	1.18 (2.19)	
Sadness	3.39 (3.33)	
Serenity	5.40 (2.57)	

*p-value was statistically significant according to Bonferroni’s correction

In comparison to probands, nonproband participants displayed higher levels of anger (*p* = 0.001), anxiety (*p* = 0.002), concern (*p* = 0.001), discomfort (*p* = 0.001) and sadness (*p* = 0.003), and they displayed both less confidence (*p* = 0.002) and serenity (*p* = 0.001). Nondiseased participants displayed higher levels of anger (*p* = 0.002), concern (*p* = 0.003), discomfort (*p* = 0.004) and sadness (*p* = 0.001) and less serenity (*p* = 0.001) than cancer patient participants. Being a carrier/noncarrier, being or not under cancer treatment, age, education, marital status or having children were found to be associated with none of the ten considered emotions.

## Discussion

The intrinsic uncertainty of genetic tests, the personal and familiar meaning given to positive or negative results, and the meaning given to cancer are but a few of the reasons that determine the emotional impact of the genetic test of susceptibility to breast/ovarian cancer and the need for further studies on this subject, as it is evident from the abundant literature on this topic [32–35] that does not provide univocal results.

The present study investigated the emotional state of women after having received the results of a genetic test for the identification of mutation on BRCA1/2 genes, through three different instruments to obtain a more comprehensive picture of emotional reactions. Moreover, in order to identify the subpopulation with the highest risk of emotional suffering, the associations of the reported emotional variables with the main socio-demographic and clinical factors were studied.

First, the data presented support the need for a psychoemotional monitoring of individuals receiving the genetic test; in addition, they increase the need for a more articulated reporting of psychological distress. In our sample, in fact, one participant out of five was a probable case of anxiety, while two participants out of five were possible cases of anxiety (using less restrictive criteria). Nearly one out of four was a possible or probable case for depression. Moreover, more than one participant out of four showed anger-hostility that exceed normative data and more than three in ten showed fatigue-inertia. Anxiety, as the HADS definition, resulted in being more intense than depression, while of the six mood states reported by POMS the most intense was fatigue-inertia. Among the ten emotions registered by the “emotions Thermometers,” the most intense were confidence and serenity, two positive emotions. Taking all things into consideration, these data may result in being contradictory; however, it must be remembered that they were obtained with instruments with different sensitivity. The apparent contradiction could be explained by the emotional constraint due to having started a genetic counseling process; for the same reason, even the low levels of confusion both in the emotional Thermometers and in POMS can be interpreted. Data on depression and anxiety evidenced that, after receiving the outcome of the test, anxiety states were bigger than depression.

Second, in accordance with some previous studies [3, 36, 37] the data presented did not report statistically significant differences attributable to the test result; in other words, women who had received a positive result (that is, who discovered they had a genetic predisposition to developing breast/ovarian cancer) did not show in the short term higher levels of negative emotions and mood states than women who had received a negative outcome. On the other hand, being/not being a proband and being or not an oncological patient (variables overlapping in 94.5% of our sample) were associated with all three types of psychological variables reported.

Compared to probands, nonprobands showed higher levels of anxiety, depression (psychological distress components) and anger-hostility; this pattern was also confirmed by data reported by the Thermometers: nonprobands showed higher levels of anger, anxiety, concern, discomfort and sadness (all emotions linked to psychological distress) [38], and less confidence and serenity. For future research, it might be interesting to allow open-ended responses from nonprobands to try to determine the source of their anxiety. In fact, it is possible that their anxiety could be related to the cancer treatments/fear of loss for their loved ones, rather than being related to genetic testing for BRCA1/2 mutations. Also comparing pre and post testing emotional states could be useful in indebting the present data.

In the present sample, the subgroup without a history of disease showed higher levels of anxiety and depression than the participants who were also oncological patients; analogously, the first showed higher levels of depression-dejection and anger-hostility than oncological patients. These data are not in accordance with what emerged in other studies [39, 40] reporting that it was women affected by cancer, especially if they have been recently diagnosed, that experienced bigger emotional suffering due to the test and/or its result and that might need greater psychological support.

Finally, in the Thermometers, women with no experience of disease showed a higher level of anger and emotions linked to the distress construct (i.e. concern, discomfort, sadness) and less serenity. In other words, women who were solicited to join genetic counseling by a proband and non-oncological patients in this sample were more vulnerable from a psychological point of view. This phenomenon can be explained in several ways. It is plausible that the probands, before deciding to join counseling, have carefully considered the pros and cons, while nonprobands, involved only later in counseling due to a genetic mutation found in the family, may have had a more careless approach. It is also likely that probands and patients during the course of their disease have come into contact with professional psychologists and eventually benefit from their support. Nonprobands and patients also have to face a family history that impacts on psychological well-being and that could lead them to consider the probability of contracting the disease not on the basis of the real risks but on what is cognitively available (heuristic availability) [41].

Based on these considerations, it would be interesting in the future to use an instrument to detect cancer-related worries to verify whether there is, in nonproband, disease-free subjects, who seem to be the most vulnerable, a high perception of the possibility of contracting the disease, regardless of the test results.

In interpreting the present data it must be borne in mind that the small size of the sample demands caution in generalizing the data, as well as the results, on emotions (i.e. collected through ad hoc built Thermometers and not through advanced instruments) and need further validation.

## Conclusion

Although limited, the present study emphasizes the clinical relevance of the emotional screening, investigates additional emotional dimensions that are usually neglected (i.e. anger), explores not only negative emotions connected to the counseling period but also the positive ones, and suggests paying particular attention to a subpopulation (nonprobands, disease-free individuals) who, contrary to what is usually believed, may be particularly vulnerable to emotional suffering.

## References

[1] AIRTUM. I numeri del cancro in Italia. Retrieved from http://www.registri-tumori.it/PDF/AIOM2014/I_numeri_del_cancro_2014.pdf

[2] NCCN. Clinical Practice Guidelines in Oncology - version 2.2014. Genetic/familial high-risk assessment: breast and ovarian. Retrieved from http://www.nccn.org/professionals/physician_gls/pdf/genetics_screening.pdf

[3] Brédart A, Kop JL, Depauw A (2013). Short-term psychological impact of the BRCA1/2 test result in women with breast cancer according to their perceived probability of genetic predisposition to cancer. Br J Cancer.

[4] Metcalfe KA, Finch A, Poll A (2009). Breast cancer risks in women with a family history of breast or ovarian cancer who have tested negative for a BRCA1 or BRCA2 mutation. Br J Cancer.

[5] van Dijk S, Timmermans DRM, Meijers-Heijboer H, Tibben A, van Asperen CJ, Otten W (2006). Clinical characteristics affect the impact of an uninformative DNA test result: the course of worry and distress experienced by women who apply for genetic testing for breast cancer. J Clin Oncol.

[6] Frank TS, Deffenbaugh AM, Reid JE (2002). Clinical characteristics of individuals with germline mutations in BRCA1 and BRCA2: analysis of 10,000 individuals. J Clin Oncol.

[7] Gadzicki D, Evans DG, Harris H (2002). Genetic testing for familial/hereditary breast cancer-comparison of guidelines and recommendations from the UK, France, the Netherlands and Germany. J Community Genet.

[8] Chen S, Parmigiani G (2007). Meta-analysis of BRCA1 and BRCA2 penetrance. J Clin Oncol.

[9] MacDonald D, Sarna L, Weitzel JN, Ferrel B (2010). Women’s perceptions of personal and family impact of genetic risk assessment: focus group findings. J Genet Counsel.

[10] Sommaggio P (2010). La consulenza genetica: Un ponte tra autopoiesi ed auto trascendimento [Genetic counseling: A bridge between autopoiesis and self-transcendence]. Tigor.

[11] Burton-Chase AM, Gritz ER, Peterson SK. Counseling e testing genetico per cancri ereditari: Considerazioni psicosociali [Genetic counseling and testing for hereditary cancers: psychosocial considerations). In: Biondi M, Costantini A, Wise TN, Eds. Psiconcologia. Milano: Raffaello Cortina Editore; 2014, 159-185.

[12] Roussi P, Sherman KA, Miller SM (2011). The identification of cognitive profiles among women considering BRCA1/2 testing through the utilization of cluster analytic techniques. Psych Health.

[13] Dorval M, Bouchard K, Maunsell E (2008). Health behaviors and psychological distress in women initiating BRCA1/2 genetic testing: comparison with control population. J Genet Counsel.

[14] Erblich J, Bovbjerg DH, Valdimarsdottir HB (2000). Looking forward and back: distress among women at familial risk for breast cancer. Ann Behav Med.

[15] Butow P, Meiser B, Price M, Bennett B, Tucker K, Davenport T, Hickie I (2005). Psychological morbidity in women at increased risk of developing breast cancer: a controlled study. Psychooncology.

[16] Coyne JC, Benazon NR, Gaba CG, Calzone K, Weber BL (2000). Distress and psychiatric morbidity among woman from high-risk breast and ovarian cancer families. J Consulting Clin Psych.

[17] Graves KD, Vegella P, Poggi EA (2012). Long-term psychosocial outcomes of BRCA1/BRCA2 testing: Differences across affected status and risk-reducing surgery choice. Cancer Epidemiol Biomarkers Prev.

[18] van Oostrom I, Meijers-Heijboer H, Lodder LN (2000). Long-term psychological impact of carrying a BRCA1/2 mutation and prophylactic surgery: a 5-year follow-up study. J Clin Oncol.

[19] Lerman C, Seay J, Balshem A, Audrain J (1995). Interest in genetic testing among first-degree relatives of breast cancer patients. Am J Med Genet.

[20] Dorval M, Patenaude AF, Schneider KA (2000). Anticipated versus actual emotional reactions to disclosure of results of genetic tests for cancer susceptibility: findings from p53 and BRCA1 testing programs. J Clin Oncol.

[21] Croyle RT, Smith KR, Botkin JR, Baty B, Nash J (1997). Psychological responses to BRCA1 mutation testing: preliminary findings. Health Psychol.

[22] Di Prospero LS, Seminsky M, Honeyford J (2001). Psychosocial issues following a positive result of genetic testing for BRCA1 and BRCA2 mutations: findings from a focus group and a needs-assessment survey. Can Med Assoc J.

[23] Vodermaier A, Esplen MJ, Maheu C (2010). Can self-esteem, mastery and perceived stigma predict long-term adjustment in women carrying a BRCA1/2- mutation? Evidence from a multi-center study. Familial Cancer.

[24] Esplen MJ, Stuckless N, Hunter J (2009). The BRCA Self-Concept Scale: a new instrument to measure self-concept in BRCA1/2 mutation carriers. Psychooncology.

[25] Pearman T, Hansen NM (2013). Psychological implications of testing positive for the BRCA gene. Management of the patient at high risk for breast cancer.

[26] Power T, Robinson JW, Bridge P, Bernier FP, Gilchrist DM (2011). Distress and psychosocial needs of a heterogeneous high risk familial cancer population. J Genet Counsel.

[27] Zigmond AS, Snaith RP (1983). The hospital anxiety and depression scale. Acta Psychiat Scand.

[28] McNair DM, Lorr M, Droppleman LF. EITS Manual for the Profile of Mood States. San Diego: Educational and Industrial Testing Service. p. 971.

[29] Wodermaier A, Linden W, Siu C (2009). Screening for emotional distress in cancer patients: A systematic review of assessment instruments. J Nat Cancer Inst.

[30] Muzzatti B, Annunziata MA (2012). Psychological distress screening in cancer patients: Psychometric properties of tools available in Italy. Tumori.

[31] Annunziata MA, Muzzatti B, Altoè G (2011). Defining Hospital Anxiety and Depression Scale (HADS) structure by confirmatory factor analysis: a contribution to validation for oncological settings. Ann Oncol.

[32] Nelson HD, Pappas M, Zakher B, Mitchell JP, Okinaka-Hu L, Fu R (2014). Risk assessment, genetic counseling, and genetic testing for BRCA-related cancer in women: A systematic review to update the U.S. Preventive Services Task Force recommendation. Ann Inter Med.

[33] Hamilton JG, Lobel M, Moyer A (2009). Emotional distress following genetic testing for hereditary breast and ovarian cancer: a meta-analytic review. Health Psych.

[34] Heshka JT, Palleschi C, Howley H, Wilson B, Wells PS (2009). A systematic review of perceived risks, psychological and behavioral impacts of genetic testing. Genet Med.

[35] Schlich-Bakker KJ, ten Kroode HFJ, Ausems MG (2006). A literature review of the psychological impact of genetic testing on breast cancer patients. Patient Educ Counsel.

[36] Bosch N, Junyent N, Gadea N, et al. What factors may influence psychological well being at three months and one year post BRCA genetic result disclosure. Breast 2012;21:755-60. 10.1016/j.breast.2012.02.004 22381151

[37] Bennet P, Wilkinson C, Turner J (2008). Psychological factors associated with emotional responses to receiving genetic risk information. J Genet Couns.

[38] NCCN. Clinical Practice Guidelines in Oncology - version 2.2014. Distress management. Retrieved from http://www.nccn.org/professionals/physician_gls/pdf/distress.pdf

[39] van Roosmalen MS, Stalmeier PF, Verhoef LC (2004). Impact of BRCA1/2 testing and disclosure of a positive test result on women affected and unaffected with breast or ovarian cancer. Am J Med Genet.

[40] Wood ME, Mullineaux L, Rahm AK, Fairclough D, Wenzel L (2000). Impact of BRCA1 testing on women with cancer: a pilot study. Genet Test.

[41] Vos J, Stiggelbout AM, Oosterwijk J (2011). A counselee-oriented perspective on risk communication in genetic counseling: explaining the inaccuracy of the counselees' risk perception shortly after BRCA1/2 test result disclosure. Genet med.

